# 

**Published:** 1868-10-01

**Authors:** 


Vol. XXV. Number 19.

THE

### Chicago Medical Journal.

*PUBLISHED SEMI-MONTHLY.*

J. Adams All n, M.D., LL.D.
EDITOR.

Walter Hay, A.M., M.D.
ASSOCIATE EDITOR.

OCTOBER 1, 1868.

CHICAGO:

Church, Goodman and Donnelley, Steam Book and Job Printers,

108 and 110 Dearborn Street.

